# Self-ordered nanospike porous alumina fabricated under a new regime by an anodizing process in alkaline media

**DOI:** 10.1038/s41598-021-86696-z

**Published:** 2021-03-31

**Authors:** Mana Iwai, Tatsuya Kikuchi, Ryosuke O. Suzuki

**Affiliations:** Division of Materials Science and Engineering, Faculty of Engineering, Hokkaido University, N13-W8, Kita-ku, Sapporo, Hokkaido 060-8628 Japan

**Keywords:** Synthesis and processing, Corrosion

## Abstract

High-aspect ratio ordered nanomaterial arrays exhibit several unique physicochemical and optical properties. Porous anodic aluminum oxide (AAO) is one of the most typical ordered porous structures and can be easily fabricated by applying an electrochemical anodizing process to Al. However, the dimensional and structural controllability of conventional porous AAOs is limited to a narrow range because there are only a few electrolytes that work in this process. Here, we provide a novel anodizing method using an alkaline electrolyte, sodium tetraborate (Na_2_B_4_O_7_), for the fabrication of a high-aspect ratio, self-ordered nanospike porous AAO structure. This self-ordered porous AAO structure possesses a wide range of the interpore distance under a new anodizing regime, and highly ordered porous AAO structures can be fabricated using pre-nanotexturing of Al. The vertical pore walls of porous AAOs have unique nanospikes measuring several tens of nanometers in periodicity, and we demonstrate that AAO can be used as a template for the fabrication of nanomaterials with a large surface area. We also reveal that stable anodizing without the occurrence of oxide burning and the subsequent formation of uniform self-ordered AAO structures can be achieved on complicated three-dimensional substrates.

## Introduction

Owing to the highly ordered periodicity of high-aspect ratio nanoscale pore arrays formed over large-scale areas, AAO is currently widely investigated and used in various nanoapplications^1^, such as plasmonic devices^2–4^, photonic crystals^5–7^, energy conversion materials^8^, high-density capacitor applications^9^, and superplastic materials^10^. An ordered porous AAO structure can be easily fabricated by anodizing Al in a few appropriate acidic electrolyte solutions, such as sulfuric, oxalic, and phosphoric acids^2,11–13^. Moreover, several additional acidic electrolytes, such as selenic, arsenic, and etidronic acids, have been recently applied for the formation of ordered porous AAOs^14–19^. However, the interpore distance (cell size) that determines the periodicity of the pore arrangement is still limited to a narrow range because ordered porous AAO structures are formed under specific operating conditions for each electrolyte. Therefore, the structural controllability of porous AAOs for many nanoapplications is also limited.

A novel technical approach known as hard anodizing (HA) has been developed to expand the interpore distance, and this technique allows the ultrarapid formation of ordered porous AAO structures with an expanded interpore distance^20–22^. However, the HA method requires a complicated cooling system to be placed under the rear surface of the specimen to remove the Joule heat generated by the rapid electrochemical reaction, and the possible anodizing area is limited to an extremely small planar region without any three-dimensional surface.

Herein, we report a novel method to fabricate an ordered porous AAO structure possessing a wide range of interpore distances and a unique spike nanomorphology by anodizing AAO in a new alkaline electrolyte, sodium tetraborate (Na_2_B_4_O_7_) aqueous solution. We demonstrate that this alkaline electrolyte allows the self-ordering of porous AAO structures over a wide nanoscale range under a new anodizing regime. We reveal that stable anodizing process without the occurrence of oxide burning and the subsequent formation of uniform ordered AAO structures can be achieved on complicated three-dimensional substrates. Finally, we also reveal that numerous vertical nanospike pores, which are completely different from conventional pores, are hexagonally arranged in the AAO film, and this ordered nanospike pore array can be applied for fabricating nanomaterials with a large surface area. Given the many advantages of the dimensions and morphologies of highly ordered porous AAO structures and the anodizing process stability, our novel alkaline electrolyte expands the basic and industrial applications of self-ordered AAO technology.

## Results and discussion

### Self-ordering of porous AAO structures during the anodizing process in alkaline sodium tetraborate solution

**Figure 1:**
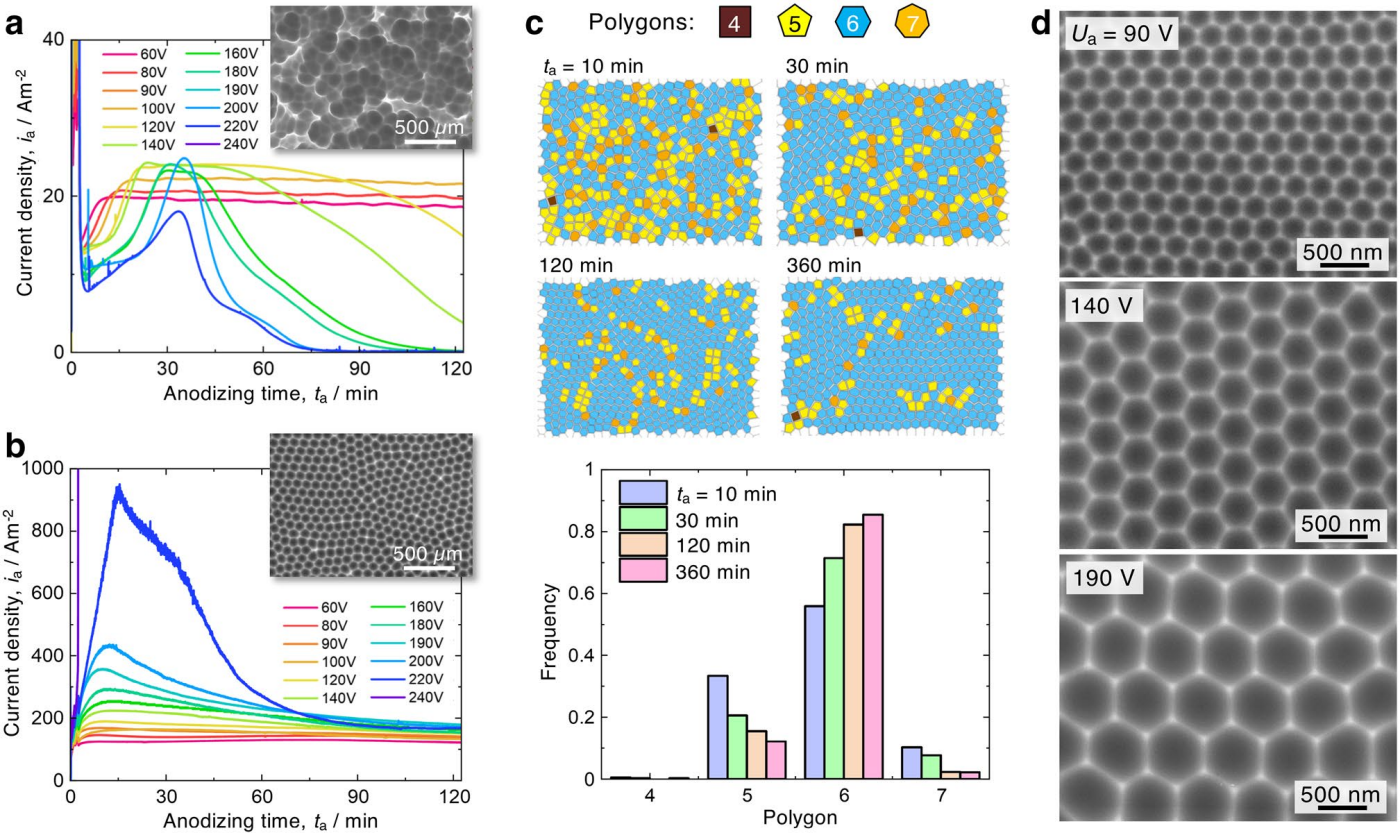
Self-ordering of porous AAO structures fabricated in alkaline Na_2_B_4_O_4_ solution. (**a**) and (**b**), Changes in the current density, *i*_a_, with the anodizing time, *t*_a_, during the potentiostatic anodizing process of Al at various applied voltages ranging from 60–240 V in a 0.5 M sodium tetraborate solution at 335 K (pH = 9.1, **a**) and 355 K (pH = 8.8, **b**). To evaluate the regularity of the porous structure, SEM images of the growth interface after anodizing at 140 V for 120 min and subsequent oxide removal are shown in each inset. (**c**) Polygonal maps (tetragon: brown, pentagon: yellow, hexagon: light blue, and heptagon: orange) of the AAO cell structure formed by anodizing at 355 K and 140 V for 10 min to 360 min and the corresponding frequencies of polygons for each anodizing time. (**d**) SEM images of the self-ordered cell structure obtained by anodizing for 360 min under the optimal conditions: concentration, 0.5 M; temperature, 355 K; and applied voltage, 90–190 V.

The changes in the nanomorphology of porous AAO structures under various anodizing conditions in alkaline sodium tetraborate solution were investigated via electrochemical measurements, scanning electron microscopy (SEM), and image analysis. The conventional anodizing method using acidic solutions for the formation of ordered porous AAO structures is frequently carried out at low temperatures to remove the generated Joule heat and induce the rapid growth of anodic oxides without “burning”^23–25^. However, extremely low current densities measuring approximately 20 Am^−2^ were encountered when the electropolished Al was potentiostatically anodized in a 0.5 M sodium tetraborate solution at less than 335 K (Fig. 1a), and unstable anodizing behaviors with a sudden drop in current density, with a value that decreased to zero, were observed at higher applied voltages. Similar electrochemical behavior is also observed in malic acid and citric acid solutions with a relatively low solubility of aluminum oxide^26^. In the low-temperature case, extremely irregular cell arrangements with a large height difference were formed at the growth interface of the anodic oxide (inset SEM image). In contrast, stable anodizing behaviors were successfully achieved at a higher temperature of 355 K (Fig. 1b). The current density gradually increased with the applied voltage due to the rapid growth of anodic oxides during the anodizing process, and the thickness of the anodic oxide may depend only on the current density measured during anodizing^27,28^. A regular honeycomb arrangement was formed at the growth interface via this high-temperature anodizing process. However, an excess applied voltage of 220 V caused a sudden increase in the current density during the early stage due to burning, and a visible nonuniform grayish oxide could be observed on the surface after the anodizing process. Although the conventional anodizing method is seldom applied under these high-temperature conditions, stable alumina dissolution for the formation of porous AAOs is induced at the bottom of pores by anodizing at high temperatures in weak alkaline solutions.

Typically, the self-ordering of the porous AAO structures progresses as the anodizing time and corresponding film thickness increase. The self-ordering behaviors were analyzed using polygonal maps of each cell structure obtained by the anodizing process at 140 V and 355 K (Fig. 1c). Because the porous AAO film formed in the early stage dissolves in the solution at high temperature of 355 K during long-term anodizing, the effect of the anodizing time on the self-ordering behavior was investigated. Various polygonal cells from tetragons to heptagons were distributed on the Al surface after the first 10 min of anodizing, and the porous AAO structures were clearly disordered after a short anodizing process. The number of hexagonal cells increased with anodizing time, and the distribution of hexagonal cells was the most uniform at more than 85% after anodizing for 360 min due to the self-ordering of the porous AAO structures. We examined the self-ordering behaviors at various applied voltages, and the self-ordering of the porous AAO structures progressed over a wide applied voltage range of 90–190 V and an interpore distance of 260–590 nm, which is a range never reported before with the conventional anodizing method (Fig. 1d)^1^. We found that there were similar ordering tendencies in various concentrations of sodium tetraborate solution at temperatures higher than 345 K, whereas no ordering behavior was observed at temperatures less than 70 V and at voltages greater than 200 V during the whole anodizing process (Supplementary Figs. S1–S7). As the temperature increased to 360 K, a nonuniform oxide film formed due to the active dissolution of the anodic oxide and the Al substrate during the anodizing process (Supplementary Fig. S8). Note that the self-ordering of the porous AAO structure formed in sodium tetraborate solution can be achieved in the middle voltage range (90–190 V) instead of at the maximum voltage, which is directly below the burning voltage observed via the conventional anodizing process (i.e., under mild conditions). This advantage is discussed in detail later for the formation of ordered porous AAO structures on complicated three-dimensional substrates.

### New regime for self-ordering porous AAO structures

**Figure 2:**
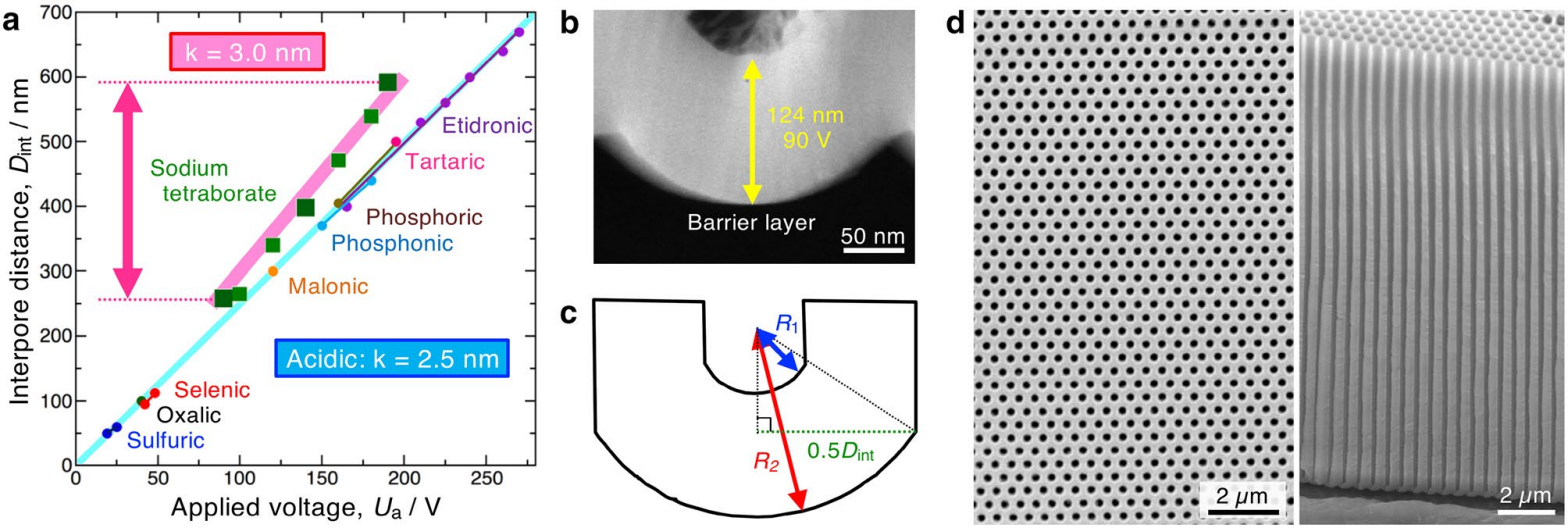
Ideally ordered porous AAO structures fabricated under a new regime in an alkaline electrolyte. (**a**) Linear relationships between the applied voltage, *U*_a_, and the interpore distance of the self-ordered porous AAO structure, *D*_int_, formed by anodizing in conventional acidic electrolyte solutions, including sulfuric, oxalic, selenic, malonic, phosphonic, phosphoric, tartaric, and etidronic acids, with a proportional constant of k = 2.5 nm V^−1^ and in an alkaline electrolyte solution, sodium tetraborate, with k = 3.0 nm V^−1^. (**b**) An HAADF-STEM image of the ultrathin section of the bottom barrier layer formed by anodizing at 90 V. A larger anodizing ratio (*δ*_b_/*U*_a_) measuring 1.38 nm V^−1^ was obtained by an alkaline anodizing process. (**c**) A schematic diagram of the bottom barrier layer with geometrical parameters of *D*_int_, *R*_1_, and *R*_2_. (**d**) SEM images of the surface and fracture cross section of the ideal porous AAO structure fabricated by nanotexturing and subsequent self-ordered anodizing at 153 V.

We found that anodizing in alkaline sodium tetraborate solution provides a new regime for the formation of self-ordering porous AAO structures. Figure 2a presents the relationship between the applied voltage and the interpore distance formed using various previously reported acidic solutions and those using our sodium tetraborate solution. It is widely known that the interpore distance of self-ordered porous AAOs, *D*_int_, formed in acidic solutions such as sulfuric, oxalic, selenic, malonic, phosphonic, phosphoric, tartaric, and etidronic acids is exactly proportional to the applied voltage, *U*_a_, with a proportionality constant of k = 2.5 nm V^−1^ (light blue line)^15,16^.

1 $$D_{{\text{int}}} = {\text{k}}U_{{\text{a}}}$$

However, the porous AAOs formed in sodium tetraborate solution did not obey this conventional regime. The interpore distance formed in sodium tetraborate also had a linear relationship with the applied voltage, but the proportionality constant obviously increased by 1.2 times, with k = 3.0 nm V^−1^, under the new regime (pink line), i.e., 1.2 times larger cells were distributed in the ordered porous AAO structure.

The thickness of the barrier layer at the bottom of the anodic oxide, *δ*_b_, is closely related to the honeycomb AAO nanostructure. The anodizing ratio, which is known as the barrier layer thickness per unit of applied voltage, for typical porous AAO structures formed in acidic solutions has been reported as approximately 1.05 nm V^−1^^29^. Figure 2b shows a high-angle annular dark field scanning transmission electron microscopy (HAADF-STEM) image of an ultrathin section of the barrier layer formed in sodium tetraborate. In this case, a relatively thick barrier layer of 124 nm was formed at 90 V, and the anodizing ratio can be calculated as 1.38 nm V^−1^, which is much larger than that formed in typical acidic solutions. The thicknesses of various barrier layers were measured by STEM, and the anodizing ratios were calculated as 1.37 nm V^−1^ at 90 V and 1.32 nm V^−1^ at 190 V (average ratio: 1.35 nm V^−1^). When the geometrical morphology of a unit cell of the porous AAO structure is specified using three parameters, including *R*_1_, *R*_2_, and *D*_int_, as described in Fig. 2c^30,31^, the barrier layer thickness is represented as *R*_2_ − *R*_1_. Here, the *R*_2_/*R*_1_ value was available in a previous study as 2.8 at 80 V^30^, and we assume that the *R*_1_ value in the electrolyte solution is almost unchanged by increasing the anodizing ratio. Considering the similarity of the triangle shown by the dotted line, the geometrical interpore distance of the porous AAO structure formed under our alkaline regime is calculated to be 1.19 times larger than that formed under other conditions, which is in good agreement with the experimental value (1.2 times higher). As described later (Supplementary Fig. S12), the anodic oxide consisted of typical amorphous aluminum oxide without electrolyte borate anions, and we could not find the difference of this oxide and typical oxides. Therefore, further investigations must be required for deeper understanding of the difference of the anodizing ratio.

Ordered porous AAO structures with various interpore distances can be fabricated via two-step anodizing or nanoimprinting methods before anodizing them in sodium tetraborate. As the nanodimpled Al surface fabricated via first an anodizing process and subsequent anodic oxide dissolution was anodized once again under the same electrochemical conditions, an ordered porous AAO structure with high-aspect-ratio nanopores could be fabricated (Supplementary Fig. S9). Although the two-step anodizing approach is a very simple method, the obtained cell distribution has some defects due to incomplete self-ordering (Fig. 1c). Therefore, nanotexturing by imprinting before anodizing can be performed to avoid the formation of any defects. An ideal honeycomb AAO structure could be fabricated over the whole Al surface by nanotexturing of a hexagonal hole array and a subsequent self-ordered anodizing step (Fig. 2d and Supplementary Fig. S10).

### The anodizing process without the occurrence of oxide burning on complicated three-dimensional Al substrates

A major advantage of anodizing in sodium tetraborate is that a stable anodizing process at higher applied voltages (more than 100 V) can be achieved without the occurrence of oxide burning on complicated three-dimensional Al substrates for the formation of self-ordered porous AAO structures. During the typical anodizing process in acidic solutions such as phosphoric and etidronic acids to form ordered porous AAOs, the self-ordering process occurs under the highest current density condition at a maximum applied voltage, which is just below the burning voltage^13,25^. Therefore, the electrolyte solution should be vigorously stirred during the anodizing process to remove the Joule heat from the Al anode and avoid burning. However, when a complicated three-dimensional Al specimen with a curved or stepped surface is anodized, oxide burning is easily induced due to the difficulty of efficiently removing the Joule heat even though the electrolyte solution is vigorously stirred. Although the HA method allows the formation of a uniform AAO film at high voltages, this technique is not adaptable to three-dimensional Al surfaces because it requires the presence of a complicated cooling system on the backside of the Al electrode to remove the Joule heat^20,21^. In contrast, self-ordering during the anodizing process in our alkaline sodium tetraborate solution can be achieved over intermediate voltage ranges (Figs. 1 and 2) rather than at the maximum voltage just below the burning voltage; thus, no vigorous stirring is required for the self-ordering of porous AAO structures.

**Figure 3:**
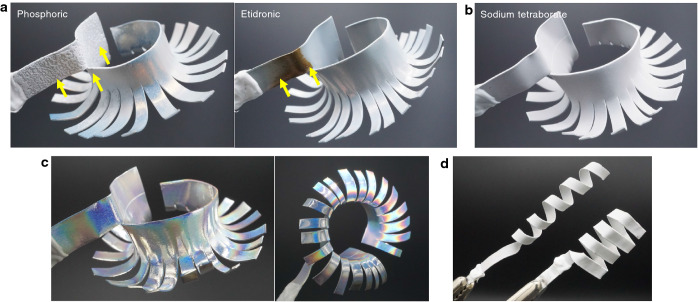
Fabrication of ordered porous AAO structures on complicated three-dimensional Al substrates. (**a**) Surface appearances of flower-shaped three-dimensional Al specimens anodized in 0.3 M phosphoric acid at 273 K and 195 V and 1.0 M etidronic acid at 303 K and 205 V. Nonuniform porous AAO films with an uneven morphology and a brown hue were partially formed on the Al surface. (**b**) Appearance of the uniform porous AAO film formed over the whole flower-shaped Al surface by anodizing in a 0.5 M sodium tetraborate solution at 355 K and 190 V. (**c**) Structural coloration based on the ordered, nanodimpled Al surface after selective dissolution of anodic oxide. (**d**) Fabrication of a self-ordered porous AAO structure without the occurrence of oxide burning on circular and square spiral Al structures.

Figure 3a, b demonstrate the self-ordered anodizing process in typical acidic solutions and in our alkaline sodium tetraborate solution using a flower-shaped three-dimensional Al specimen. The anodizing processes in phosphoric acid and etidronic acid solutions caused the formation of a nonuniform AAO film with a bumpy surface or a burned brown hue due to oxide burning (yellow arrows). In contrast, anodizing in sodium tetraborate enabled the formation of a completely uniform AAO film without the occurrence of oxide burning even on the flower-shaped three-dimensional surface. After the selective dissolution of the anodic oxide, nanostructural coloration based on the self-ordered dimple array with an average diameter of 570 nm can be observed on the whole Al surface (Fig. 3c). Our alkaline anodizing technique provides a method for the uniform formation of self-ordered porous AAO structures without the occurrence of oxide burning on complicated Al surfaces, such as circular and square spiral structures, due to the mild anodizing behavior (Fig. 3d).

### Self-ordered nanospike pore array

**Figure 4:**
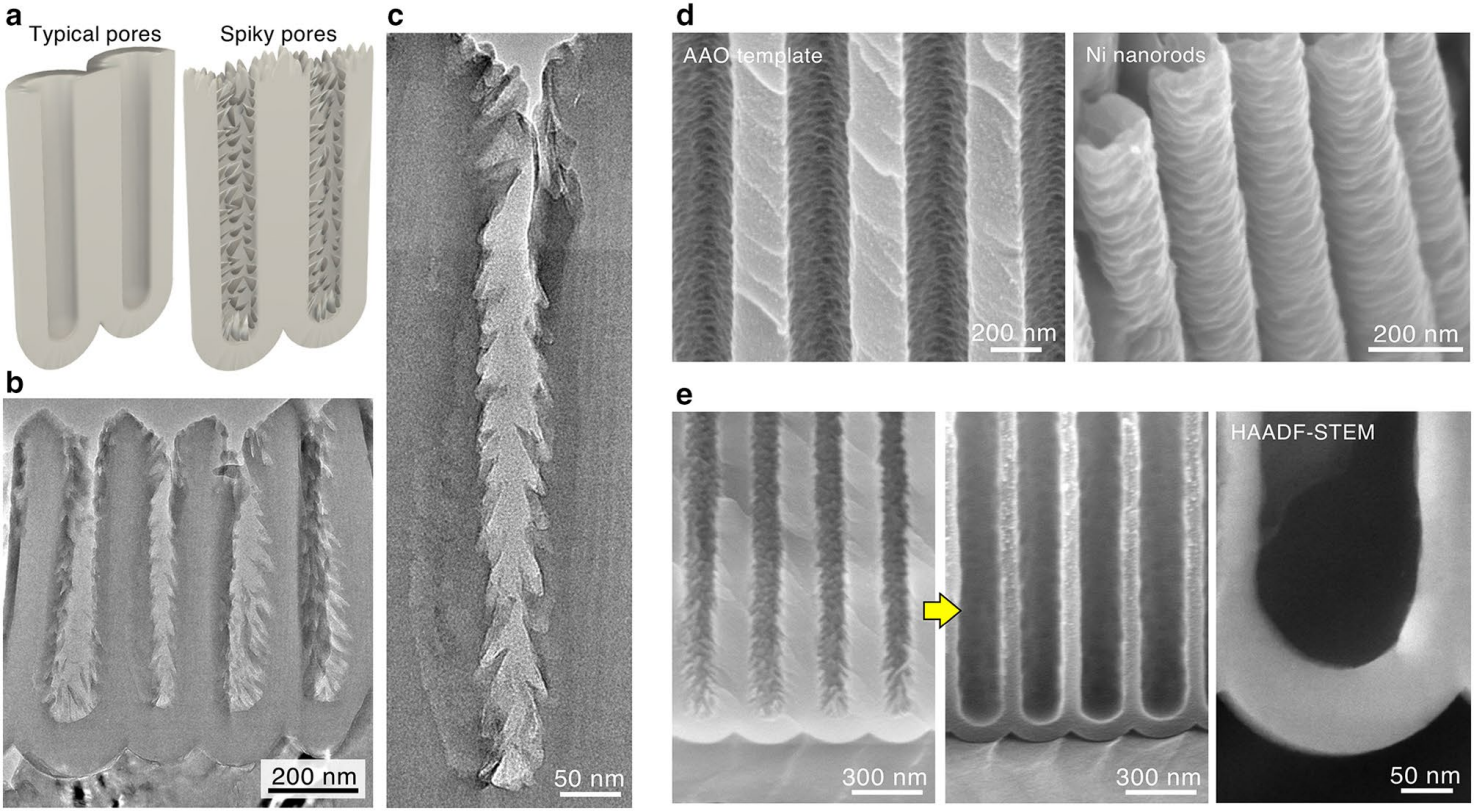
Highly ordered nanospike pore arrays. (**a**) Schematics of the typical porous AAO structure with flat, smooth cylindrical pores formed in typical acidic electrolytes and the nanospike AAO structure with numerous oxide spikes on the pore walls. (**b**) A TEM image of an ordered porous AAO structure fabricated via two-step anodizing of Al in a 0.5 M sodium tetraborate solution at 355 K and 140 V for 120 min (first anodizing process) and 10 min (second anodizing process). (**c**) A high-magnification TEM image of a nanospike pore wall from the top surface to the bottom interface of the porous layer. (**d**) SEM images of the ordered porous AAO template and corresponding Ni nanorods obtained by electroplating using the AAO template with the nanospike morphology. (**e**) Pore-widening of the porous AAO structure in a 1.0 M phosphoric acid solution at 298 K for 120 min. The nanospikes completely disappeared through pore widening.

The pore walls of typical porous AAO structures formed in acidic solutions have a flat, smooth surface; thus, complete cylindrical nanopores are hexagonally arranged in the ordered AAO structure (Fig. 4a, left)^20^. Interestingly, characteristic rugged pores with numerous nanospikes were arranged by anodizing in sodium tetraborate solution, which is different from the conventional pores (Fig. 4a, right). Figure 4b represents a TEM image of the vertical cross section of an ultrathin section of a porous AAO film formed in sodium tetraborate, and it can be seen that vertical pore walls were covered with many spikes, resembling feathers. These nanospikes were formed with a periodicity of several tens of nanometers and were present over the entire surface of the pore walls from the top surface to the bottom interface (Fig. 4c and supplementary Fig. S11). Because the anodic oxide grows at the bottom of pores, i.e., forming a barrier layer during the anodizing process due to several possible reasons including ion migration, field-assisted oxide dissolution, oxide flow, and oxygen gas evolution^30,32–35^, the spiked porous layer on the barrier layer is basically composed of residuals after anodic oxide formation. In addition, as described below, this spiked morphology was not formed via chemical dissolution in alkaline solution. Therefore, these nanospikes are considered to be generated at the bottom of pores during the anodizing process. The AAOs containing the nanospike oxides consisted of an amorphous aluminum oxide without electrolyte borate anions (Supplementary Fig. S12).

It was previously reported that similar nanospike pores could be observed in the porous AAOs formed only in chromic acid as a special case^36,37^, whereas the nanopores of the porous AAO were poorly arranged during the anodizing process under all of the operating conditions; thus, an ordered porous AAO structure could not be obtained^38,39^. Therefore, our highly ordered nanospike pore array is very useful for fabricating various nanomaterials with a large surface area. Figure 4d shows SEM images of an ordered porous AAO template and the subsequently formed Ni nanorods fabricated by electroplating using the AAO template. Many Ni nanorods with a nanoscale and bumpy surface corresponding to the concave-convex shape of the pore wall of porous AAO structures could be successfully fabricated by templating. Alternatively, a pore-widening method enabled the formation of cylindrical-shaped nanopores with a flat surface (Fig. 4e). As the porous AAO structure was immersed in phosphoric acid etching solution, the convex parts of the nanospike oxide were preferentially dissolved into the etchant, and larger pores with a flat, smooth surface could also be fabricated. This is due to the extremely narrow shape of the feathered walls, and the nanospikes rapidly disappeared in the initial stage of pore widening. Therefore, our self-ordered porous AAO structure formed via a new alkaline anodizing method enables the development of previously underutilized nanomorphological characteristics, such as the interpore distance, pore size, and surface roughness.

## Conclusions

In the present investigation, we fabricated highly ordered porous AAO structures with a nanospike hexagonal pore array by anodizing Al in a new electrolyte, namely, sodium tetraborate aqueous solution. This alkaline electrolyte operates under a new anodizing regime with a proportionality constant of 3.0 nm/V for the linear relation between the voltage and interpore distance and allows self-ordering of the pore structures with a wide interpore distance range of 260–590 nm. Ordered porous AAO structures can be successfully fabricated by two-step anodizing or by a nanoimprint technique before the anodizing process. We also demonstrated the formation of stable porous AAO structures without the occurrence of oxide burning on complicated three-dimensional Al substrates. We expect that our novel anodizing process using alkaline sodium tetraborate solution will be a useful technique for the fabrication of materials for various nanoapplications, including photoelectrodes, plasmonic devices, and photonic crystals.

## Methods

### Anodizing Al in sodium tetraborate aqueous solutions

High-purity Al plates (99.999%, 500 µm thick, Nippon Light Metal, Japan) were ultrasonically cleaned in 99.5% ethanol for 10 min. The Al specimens were immersed in a 22 vol% solution of 70% perchloric acid/78 vol% acetic acid below 280 K and then electropolished for 2 min at a constant applied voltage of 28 V using a direct power supply. A large Al plate was used as the cathode. After electropolishing, the specimens were immediately washed with ultrapure water (18.2 MΩ·cm) and were then completely dried with an air blower.

The electropolished Al specimens were anodized in 0.2–0.8 M sodium tetraborate aqueous solutions. Because these concentrations exceed the solubility limit in water at room temperature, solid sodium tetraborate powders were completely dissolved in ultrapure water at higher temperatures, and the prepared electrolyte solutions were maintained in a constant temperature incubator at 353 K until they were subjected to the anodizing process. The Al anode and Pt cathode were immersed in the electrolyte solution under stirring with a rotating speed of 500 rpm using a magnetic stir bar, and the potentiostatic anodizing process was conducted under various operating conditions at a constant applied voltage of 60–240 V and a temperature of 335–361 K for up to 360 min using a direct power supply. The applied voltage linearly increased to the assigned value in the first 2.5 min and was then maintained at a constant value. The solution temperature was maintained with a constant temperature water bath and an oil bath, and the current was recorded with a digital multimeter during the potentiostatic anodizing process. To compare the anodizing behaviors, the electropolished Al specimens were also anodized in a 0.3 M phosphoric acid solution at 273 K and 195 V and a 1.0 M etidronic acid solution at 303 K and 205 V.

After the anodizing process was applied to the specimens, Ni metal layers were electrodeposited into the nanoscale pores in the porous AAO template to fabricate Ni nanorods. The electrodeposition was carried out in a 0.12 M NiSO_4_/0.12 M NiCl_2_/0.5 M H_3_BO_3_ solution at 313 K and 200 Am^−2^ for 2 h. Because this porous AAO template has a bottom thin barrier layer, Ni metal layers were electrodeposited into the porous layer through the imperfections in the barrier layer, and non-uniform nickel deposits were obtained on the anodized surface.

### Fabrication of ordered porous AAO structures

Two-step anodizing in sodium tetraborate solution was carried out to fabricate ordered porous AAO structures. The electropolished Al specimens were anodized as mentioned above (the first anodizing process). The anodized Al specimens were immersed in a 0.2 M chromic acid/0.51 M phosphoric acid solution at 353 K, and the anodic oxide was completely dissolved to expose a nanodimpled Al surface. These Al specimens were anodized at the same applied voltages as the first anodizing process without a linear voltage increase (the second anodizing process), and ordered porous AAO structures were fabricated on the Al surface. After the two-step anodizing process was complete, pore widening was carried out via immersion of the specimens in a 1.0 M phosphoric acid solution at 298 K for up to 120 min.

A nanoimprinting technique was also conducted for the fabrication of highly ordered porous AAO structures. The Al specimen was heated at 773 K for 2 h in air to soften the Al substrate and was then electropolished. Nanoimprinting of the softened Al surface was achieved using a Si mold with a hexagonal packed array pattern of pillars with a 230 nm diameter and a 460 nm pitch. The pressure between the Al specimen and the Si master mold was applied using a hydraulic press system. Herein, a highly ordered hexagonal hole array acting as the initial sites for pore growth was fabricated on the Al surface. The nanotextured Al specimen was anodized in a 0.5 M sodium tetraborate solution at 355 K and 153 V for 10–60 min to grow highly ordered porous AAO structures.

### Ultramicrotomy

The anodized specimens were vertically embedded in an epoxy resin using embedding capsules with a truncated pyramid tip. After curing the epoxy resin, the tip of the specimens was shaved with silicon carbide grinding paper and a glass knife prepared with a glass knife maker. These pretreated Al specimens were thinned to 25–40 nm by an ultrathin sectioning method using an ultramicrotome (Powertome XL, RMC) and a diamond knife, and the ultrathin sections were floated on ultrapure water in a water container. Finally, the ultrathin specimens were collected from the water surface using a copper mesh grid with a support film.

### SEM and STEM observations

The nanomorphologies of the surface and the fractured cross section of the anodized specimens were examined by field emission (FE)-SEM (JSM6500F, JEOL). A thin electroconductive platinum film was coated on the surface of the specimen before SEM observations were performed for insulating anodic oxide. The ultrathin sectioned specimens were examined by Cs-corrected STEM (Titan3 G2 60-300, FEI). The crystallinity of the AAO was investigated by electron diffraction analysis, and elemental analysis was conducted using EDS and EELS.

### Image analysis of the porous AAO structure

Polygonal maps of the cell distribution, including triangles, tetragons, pentagons, hexagonal heptagons, and octagons, were drawn by a homemade image analysis program developed using the OpenCV library in Python. The frequency of the polygons was calculated using at least 100 cells. The interpore distance of the porous AAO structure was calculated by image analysis software (Image-Pro 10, Media Cybernetics).

## Supplementary Information

Supplementary Figures.

## References

[1] Lee W, Park SJ (2014). Porous anodic aluminum oxide: Anodization and templated synthesis of functional nanostructures. Chem. Rev..

[2] Masuda H, Fukuda K (1995). Ordered metal nanohole arrays made by a two-step replication of honeycomb structures of anodic alumina. Science.

[3] Wen L, Xu R, Mi Y, Lei Y (2017). Multiple nanostructures based on anodized aluminium oxide templates. Nat. Nanotechnol..

[4] Kondo T, Miyazaki H, Yanagishita T, Masuda H (2018). Anodic porous alumina with elliptical apertures. Electrochem. Commun..

[5] Masuda H, Yamada M, Matsumoto F, Yokoyama S, Mashiko S, Nakao M, Nishio K (2006). Lasing from two-dimensional photonic crystals using anodic porous alumina. Adv. Mater..

[6] Martín J, Martín-González M, Fernández JF, Caballero-Calero O (2014). Ordered three-dimensional interconnected nanoarchitectures in anodic porous alumina. Nat. Commun..

[7] Yanagishita T, Nishio K, Masuda H (2008). Two-dimensional photonic crystal composed of ordered polymer nanopillar arrays with high aspect ratios using anodic porous alumina. Appl. Phys. Lett..

[8] Ozel T, Bourret GR, Mirkin CA (2015). Coaxial lithography. Nat. Nanotechnol..

[9] Lee W, Han H, Lotnyk A, Schubert MA, Senz S, Alexe M, Hesse D, Bail S, Gösele U (2008). Individually addressable epitaxial ferroelectric nanocapacitor arrays with near Tb inch^−2^ density. Nat. Nanotechnol..

[10] Aramesh M, Mayamei Y, Wolff A, Ostrikov KK (2018). Superplastic nanoscale pore shaping by ion irradiation. Nat. Commun..

[11] Thompson GE, Wood GC (1981). Porous anodic film formation on aluminium. Nature.

[12] Masuda H, Hasegwa F, Ono S (1997). Self-ordering of cell arrangement of anodic porous alumina formed in sulfuric acid solution. J. Electrochem. Soc..

[13] Masuda H, Yada K, Osaka A (1998). Self-ordering of cell configuration of anodic porous alumina with large-size pores in phosphoric acid solution. Jpn. J. Appl. Phys..

[14] Nishinaga O, Kikuchi T, Natsui S, Suzuki RO (2013). Rapid fabrication of self-ordered porous alumina with 10-/sub-10-nm-scale nanostructures by selenic acid anodizing. Sci. Rep..

[15] Kikuchi T, Nishinaga O, Natsui S, Suzuki RO (2015). Fabrication of self-ordered porous alumina via etidronic acid anodizing and structural color generation from submicrometer-scale dimple array. Electrochim. Acta.

[16] Takenaga A, Kikuchi T, Natsui S, Suzuki RO (2016). Exploration for the self-ordering of porous alumina fabricated via anodizing in etidronic acid. Electrochim. Acta.

[17] Akiya S, Kikuchi T, Natsui S, Suzuki RO (2017). Nanostructural characterization of large-scale porous alumina fabricated via anodizing in arsenic acid solution. Appl. Surf. Sci..

[18] Iwai M, Kikuchi T, Suzuki RO, Natsui S (2019). Electrochemical and morphological characterization of porous alumina formed by galvanostatic anodizing in etidronic acid. Electrochim. Acta.

[19] Akiya S, Kikuchi T, Natsui S, Sakaguchi N, Suzuki RO (2016). Self-ordered porous alumina fabricated via phosphonic acid anodizing. Electrochim. Acta.

[20] Lee W, Ji R, Gösele U, Nielsch K (2006). Fast fabrication of long-range ordered porous alumina membranes by hard anodization. Nat. Mater..

[21] Lee W, Schwirn K, Steinhart M, Pippel E, Scholz R, Gösele U (2008). Structural engineering of nanoporous anodic aluminium oxide by pulse anodization of aluminium. Nat. Nanotechnol..

[22] Huang H, Qiu J, Wei X, Sakai E, Jiang G, Wu H, Komiyama T (2020). Ultra-fast fabrication of porous alumina film with excellent wear and corrosion resistance via hard anodizing in etidronic acid. Surf. Coat. Technol..

[23] Ono S, Saito M, Asoh H (2004). Self-ordering of anodic porous alumina induced by local current concentration: Burning. Electrochem. Solid State Lett..

[24] Aerts T, De Graeve I, Terryn H (2008). Study of initiation and development of local burning phenomena during anodizing of aluminium under controlled convection. Electrochim. Acta.

[25] Iwai M, Kikuchi T, Suzuki RO (2021). High-speed galvanostatic anodizing without oxide burning using a nanodimpled aluminum surface for nanoporous alumina fabrication. Appl. Surf. Sci..

[26] Kikuchi T, Yamamoto T, Suzuki RO (2013). Growth behavior of anodic porous alumina formed in malic acid solution. Appl. Surf. Sci..

[27] Zhang Z, Wang Q, Xu H, Zhang W, Zhou Q, Zeng H, Yang J, Zhu J, Zhu X (2020). TiO_2_ nanotube arrays with a volume expansion factor greater than 2.0: Evidence against the field-assisted ejection theory. Electrochem. Commun..

[28] Kikuchi T, Akiya S, Kunimoto K, Suzuki RO, Natsui S (2020). Photoluminescence from anodic aluminum oxide formed via etidronic acid anodizing and enhancing the intensity. Mater. Trans..

[29] Ebihara K, Takahashi H, Nagayama M (1983). Structure and density of anodic oxide films formed on aluminum in oxalic acid solutions. J. Metal Surf. Fin. Soc. Jpn..

[30] Houser JE, Hebert KR (2009). The role of viscous flow of oxide in the growth of self-ordered porous anodic alumina films. Nat. Mater..

[31] Hebert KR, Albu SP, Paramasivam I, Schmuki P (2012). Morphological instability leading to formation of porous anodic oxide films. Nat. Mater..

[32] Takahashi H, Nagayama M (1978). Electrochemical behaviour and structure of anodic oxide films formed on aluminium in a neutral borate solution. Electrochim. Acta.

[33] Oh J, Thompson CV (2011). The role of electric field in pore formation during aluminum anodization. Electrochim. Acta.

[34] Zhu XF, Song Y, Liu L, Wang CY, Zheng J, Jia HB, Wang XL (2009). Electronic currents and the formation of nanopores in porous anodic alumina. Nanotechnology.

[35] Li D, Zhao L, Jiang C, Lu JG (2010). Formation of anodic aluminum oxide with serrated nanochannels. Nano. Lett..

[36] Thompson GE, Furneaux RC, Wood GC (1978). Electron microscopy of ion beam thinned porous anodic films formed on aluminium. Corr. Sci..

[37] Elabar D, Němcová A, Hashimoto T, Skeldon P, Thompson GE (2015). Effect of sulphate impurity in chromic acid anodizing of aluminium. Corr. Sci..

[38] Stępniowski WJ, Michalska-Domańska M, Norek M, Czujko T (2014). Fast Fourier transform based arrangement analysis of poorly organized alumina nanopores formed via self-organized anodization in chromic acid. Mater. Lett..

[39] Stępniowski WJ, Norek M, Michalska-Domańska M, Bombalska A, Nowak-Stępniowska A, Kwaśny M, Bojar Z (2012). Fabrication of anodic aluminum oxide with incorporated chromate ions. Appl. Surf. Sci..

