# Supplementary material for: Self-ordered nanospike porous alumina fabricated under a new regime by an anodizing process in alkaline media
Source: Sci Rep. 2021 Mar 31;11:7240. doi: 10.1038/s41598-021-86696-z (PMC8012646; doi:10.1038/s41598-021-86696-z)
Supplement: Supplementary file 1 — Supplementary Figures. [file 41598_2021_86696_MOESM1_ESM.pdf]

Supplementary Information for

Self-ordered nanospike porous alumina fabricated under a new regime by an anodizing process in alkaline media

Mana Iwai, Tatsuya Kikuchi*, Ryosuke O. Suzuki

Division of Materials Science and Engineering, Faculty of Engineering, Hokkaido University, N13-W8, Kita-ku, Sapporo, Hokkaido, 060-8628, Japan

*Corresponding author: Tatsuya Kikuchi

e-mail: kiku@eng.hokudai.ac.jp

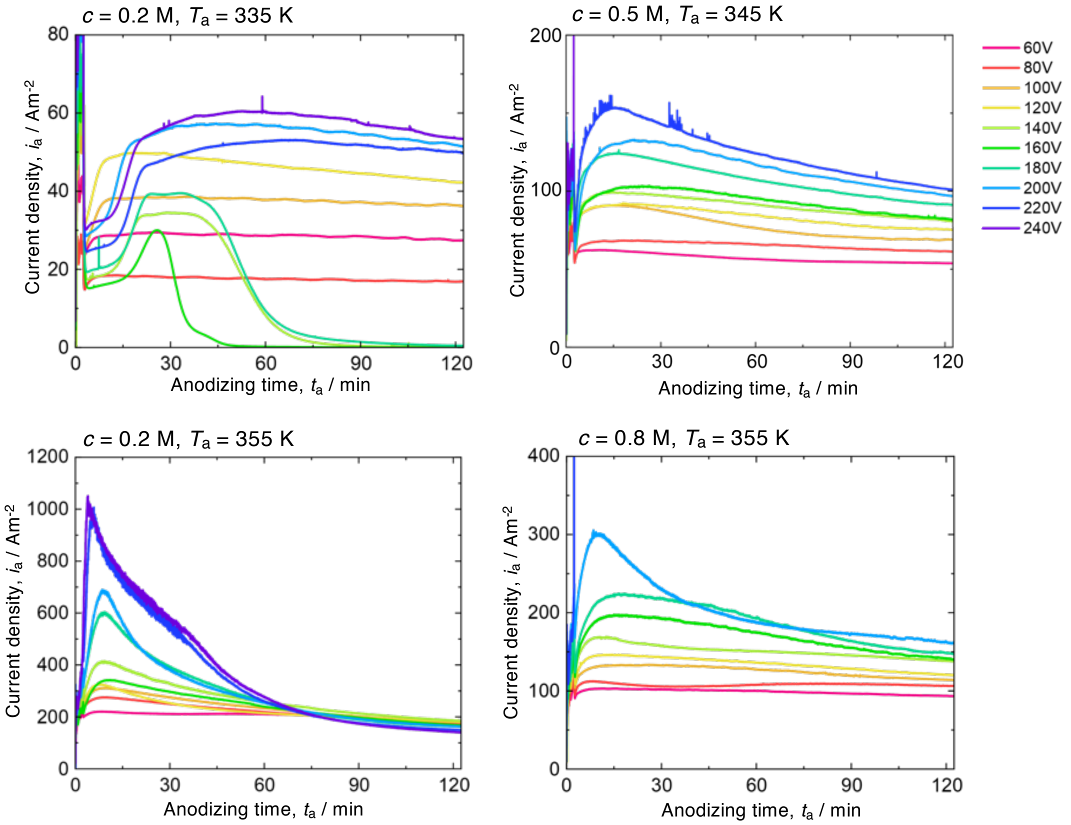

Supplementary Figure S1 Current density-time curves during the anodizing process in sodium tetraborate solutions under various operating conditions (see Fig. 1 for details of the anodizing process in a 0.5 M sodium tetraborate solution at 335 K and 355 K). The current density increased with the temperature at the same applied voltages. Self-ordering behaviors were identified at temperatures greater than 345 K under high current density conditions.

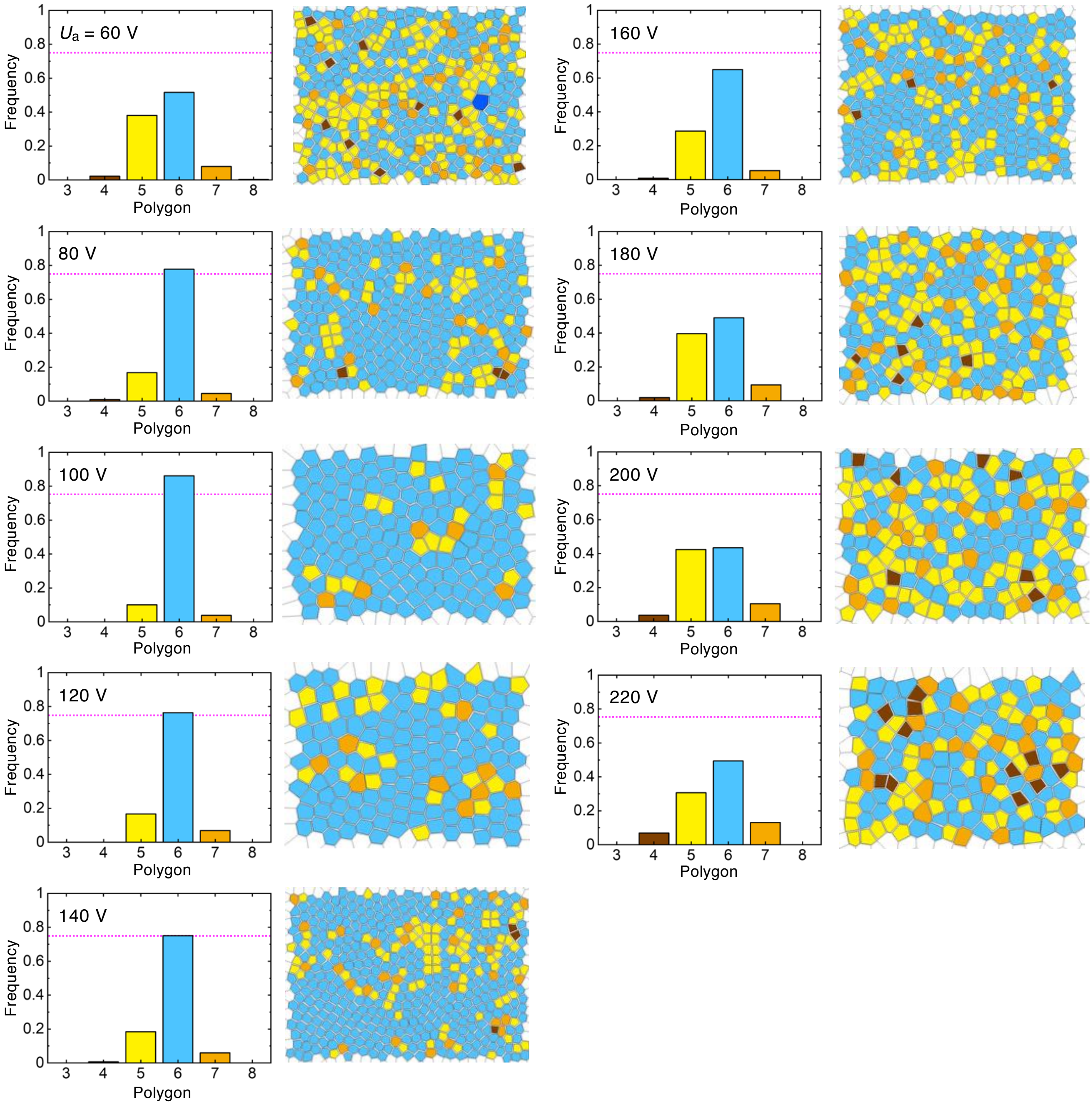

Supplementary Figure S2 Polygonal maps (tetragon: brown, pentagon: yellow, hexagon: light blue, heptagon: orange, and octagon: blue) of the AAO cell structure formed by anodizing in a 0.2 M sodium tetraborate solution at 355 K (pH = 8.7) and various applied voltages of 60-220 V for 120 min, and the corresponding frequencies of polygons at each anodizing voltage. The dotted line corresponds to 75% of the total frequency, and high regularities of the porous AAO structure were obtained at 80-140 V.

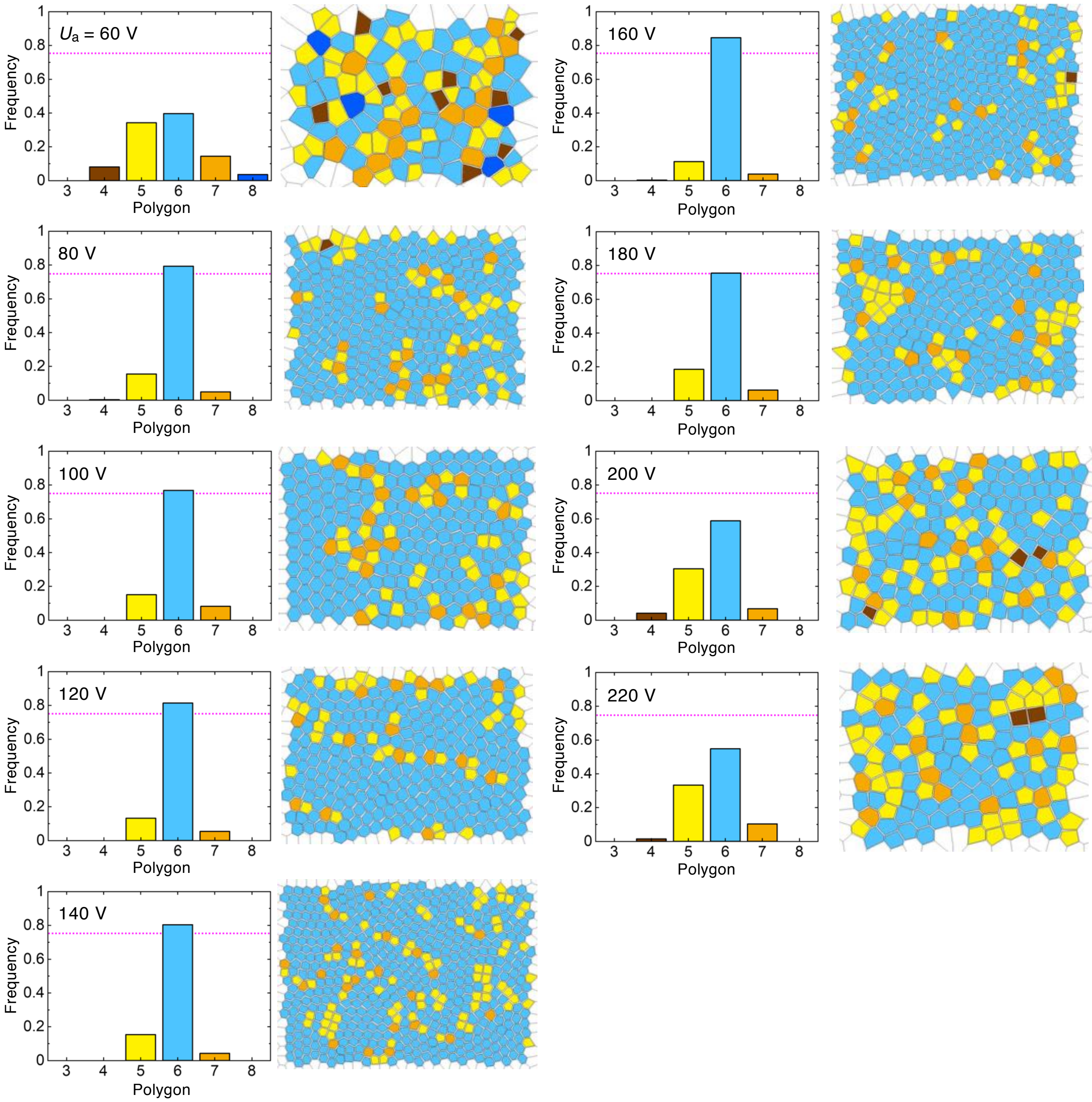

Supplementary Figure S3 Polygonal maps of the AAO cell structure formed by anodizing in a 0.5 M sodium tetraborate solution at 355 K (pH = 8.8) and various applied voltages of 60-220 V for 120 min, and the corresponding frequencies of polygons at each anodizing voltage. High regularities of the porous AAO structure were obtained at 80-180 V.

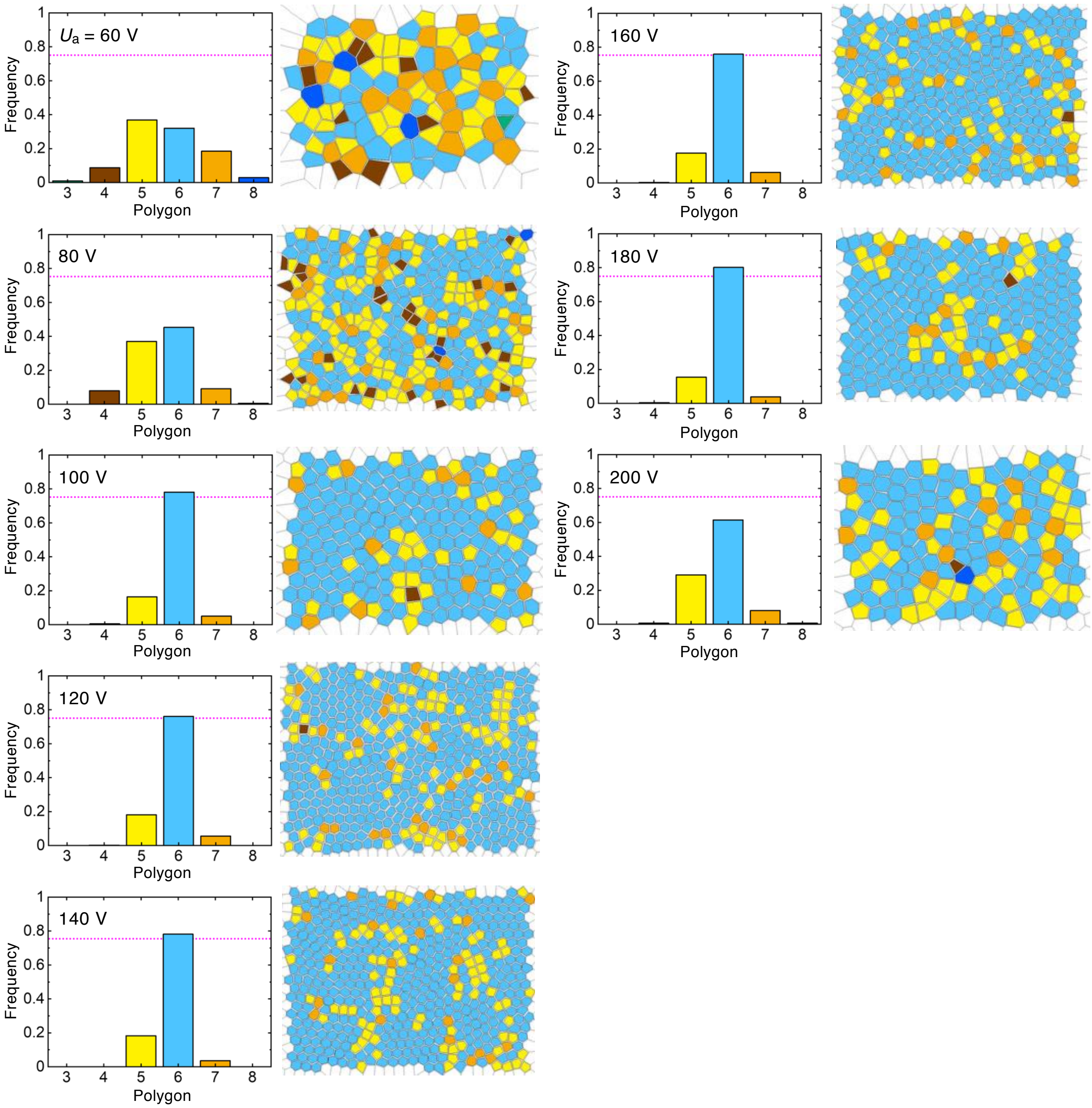

Supplementary Figure S4 Polygonal maps (triangle: green) of the AAO cell structure formed by anodizing in a 0.8 M sodium tetraborate solution at 355 K (pH = 8.8) and various applied voltages of 60-200 V for 120 min, and the corresponding frequencies of polygons at each anodizing voltage. High regularities of the porous AAO structure were obtained at 100-180 V.

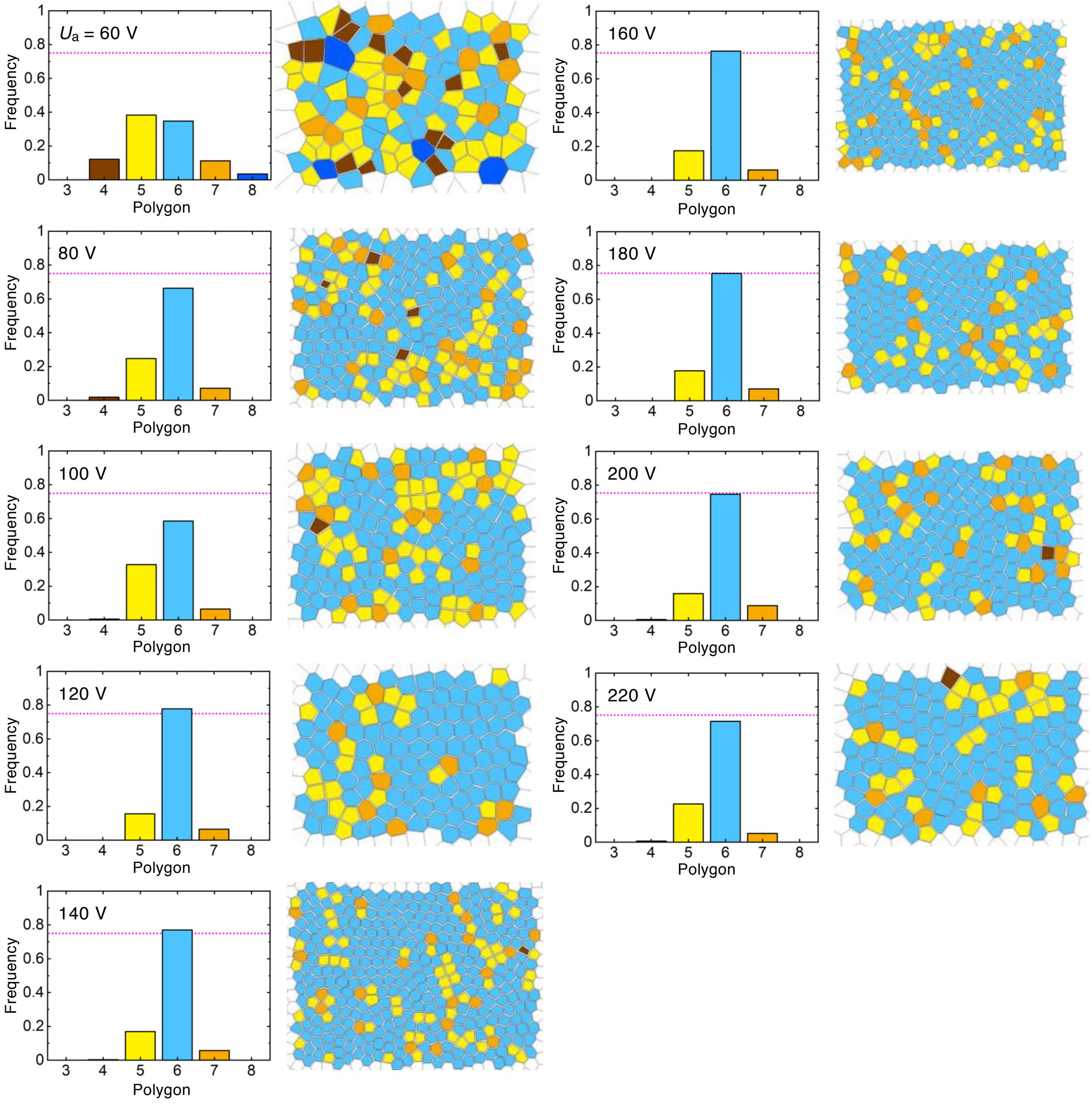

Supplementary Figure S5 Polygonal maps of the AAO cell structure formed by anodizing in a 0.5 M sodium tetraborate solution at 345 K (pH = 8.8) and various applied voltages of 60-200 V for 120 min, and the corresponding frequencies of polygons at each anodizing voltage. High regularities of the porous AAO structure were obtained at 120-180 V.

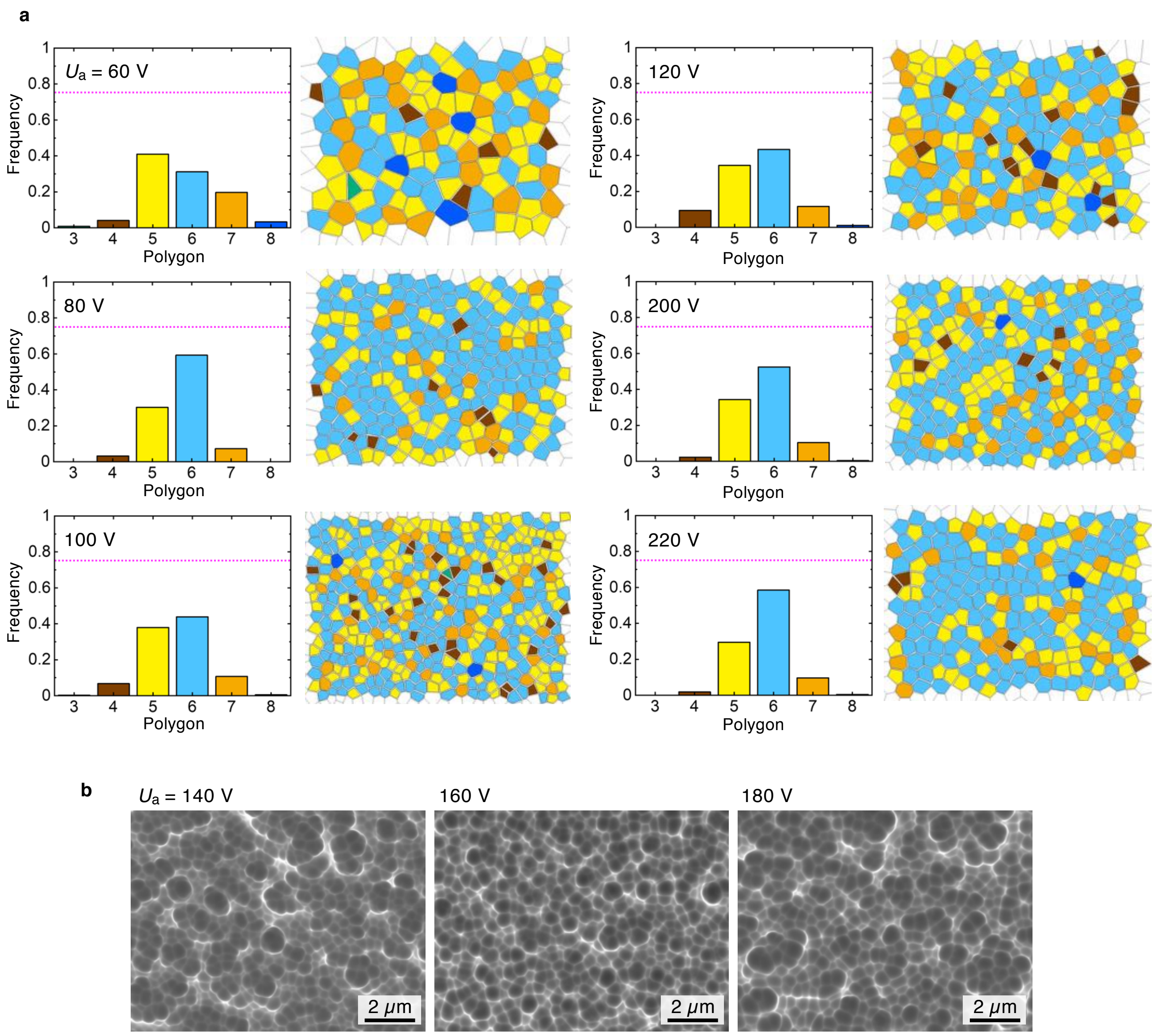

Supplementary Figure S6 a, Polygonal maps of the AAO cell structure formed by anodizing in a 0.2 M sodium tetraborate solution at 335 K (pH = 9.1) and 60-120 V/200-220 V for 120 min, and the corresponding frequencies of polygons for each anodizing voltage. Disordered structures were observed at each applied voltage. **b,** SEM images of the growth interface formed at 140-180 V under the same anodizing conditions. Extremely bumpy interfaces with a disordered cell structure were formed at these applied voltages.

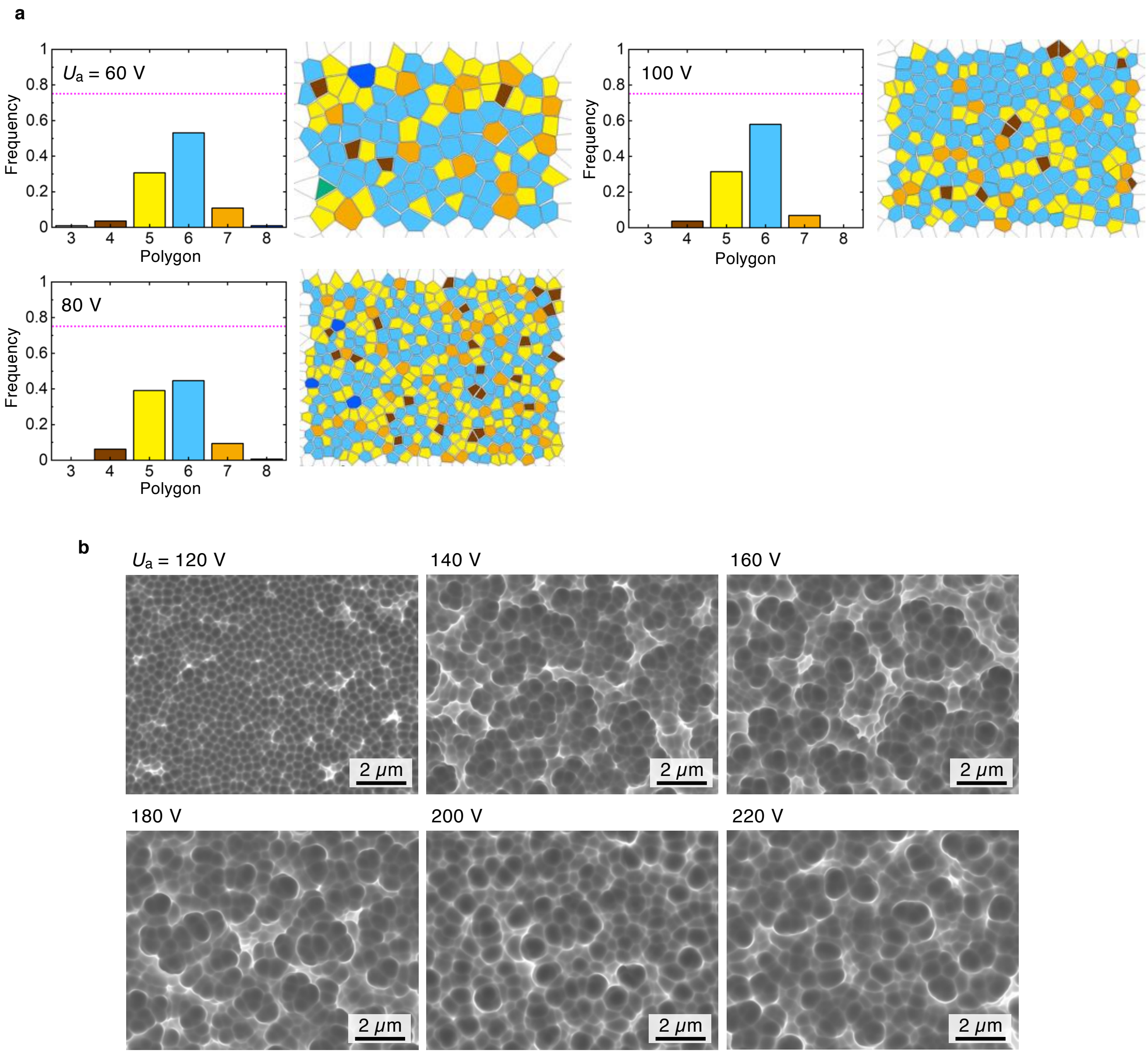

Supplementary Figure S7 a, Polygonal maps of the AAO cell structure formed by anodizing in a 0.5 M sodium tetraborate solution at 335 K (pH = 9.1) and 60-100 V for 120 min, and the corresponding frequencies of polygons for each anodizing voltage. Disordered structures were observed at each applied voltage. **b,** SEM images of the growth interface formed at 120-220 V under the same anodizing conditions. Extremely bumpy interfaces with a disordered cell structure were formed at these applied voltages.

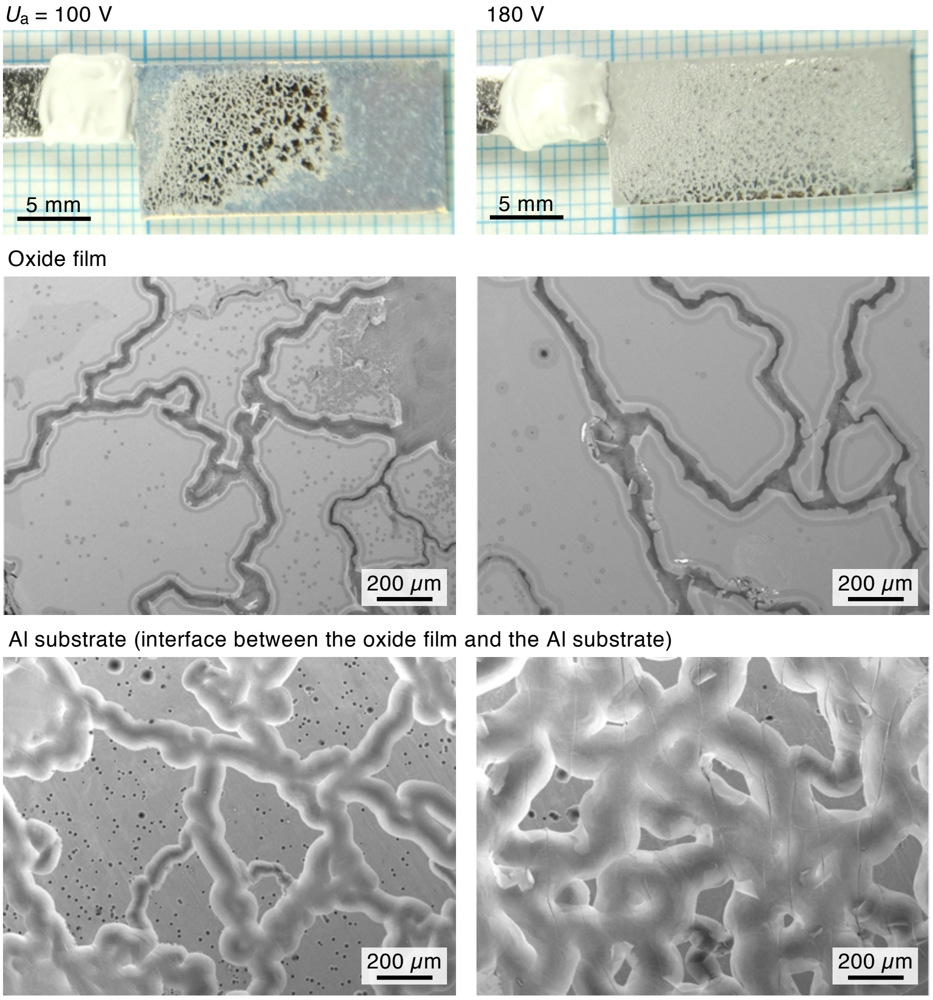

Supplementary Figure S8 Surface appearances (upper) and corresponding SEM images (middle and lower) of the Al specimens anodized in a 0.5 M sodium tetraborate solution at a high temperature of 361 K (pH = 8.7) and from 100-180 V for 120 min. Nonuniform anodic oxide films were formed on each anodized surface, and many etched lines were observed on the Al substrate due to active dissolution in the high-temperature electrolyte solution.

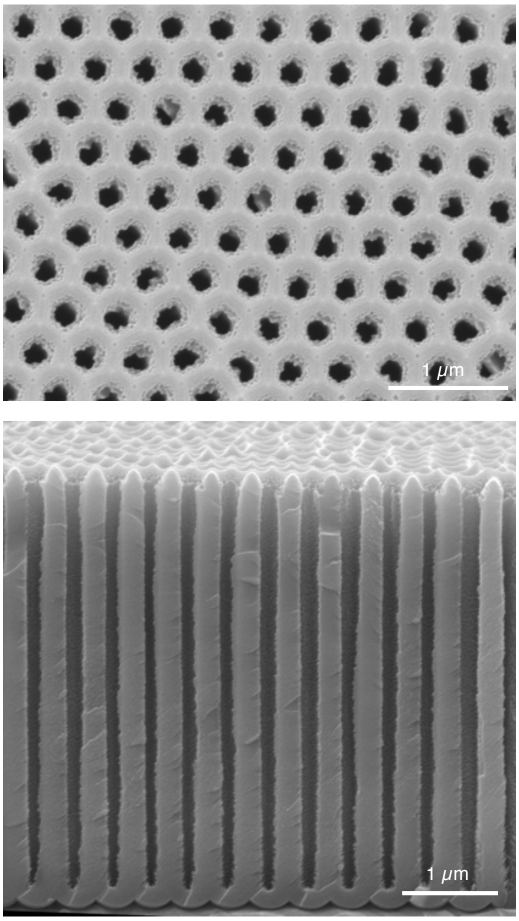

Supplementary Figure S9 SEM images of the surface and fracture cross section of the self-ordered porous AAO structure fabricated via a two-step anodizing process in a 0.5 M sodium tetraborate solution at 355 K and 140 V for 120 min (first anodizing process) and 10 min (second anodizing process).

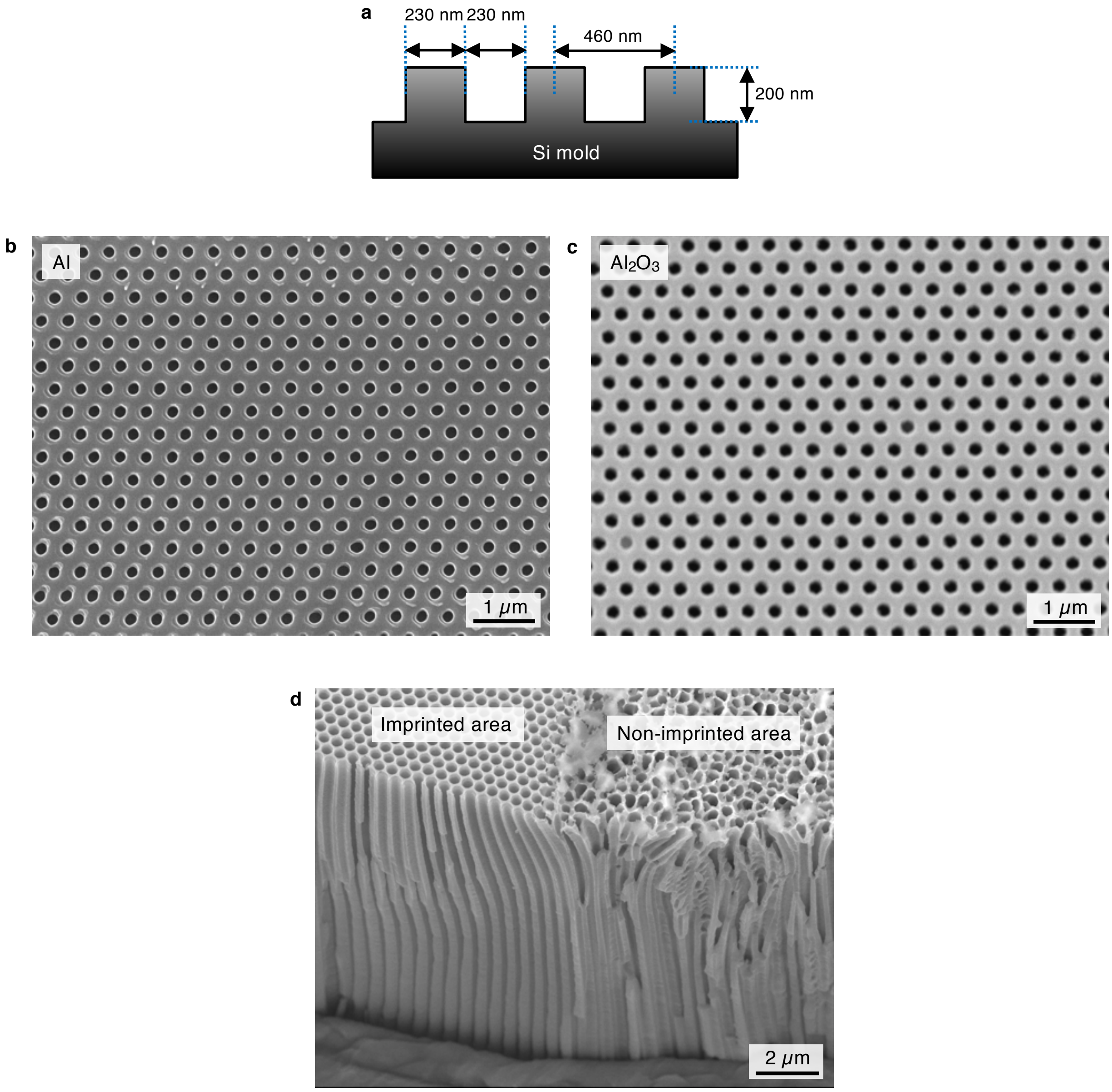

Supplementary Figure S10 a, A schematic illustration of the vertical cross section of the Si master mold with a hexagonal dot array. **b,** An SEM image of the aluminum surface after nanoimprinting. An ideal hexagonal hole array was fabricated on the Al surface. **c,** An SEM image of the surface anodized in 0.5 M sodium tetraborate at 355 K and 153 V for 10 min. An ideal porous AAO structure can be observed on the anodized surface. **d,** An SEM image of the fracture cross section of the porous AAO structure at the boundary between the imprinted area and the nonimprinted area. Disordered pores grew from the nonimprinted surface, whereas the pores were gradually rearranged during the anodizing process under self-ordering conditions. In contrast, an ordered pore structure can be observed in the imprinted area.

a

b

c

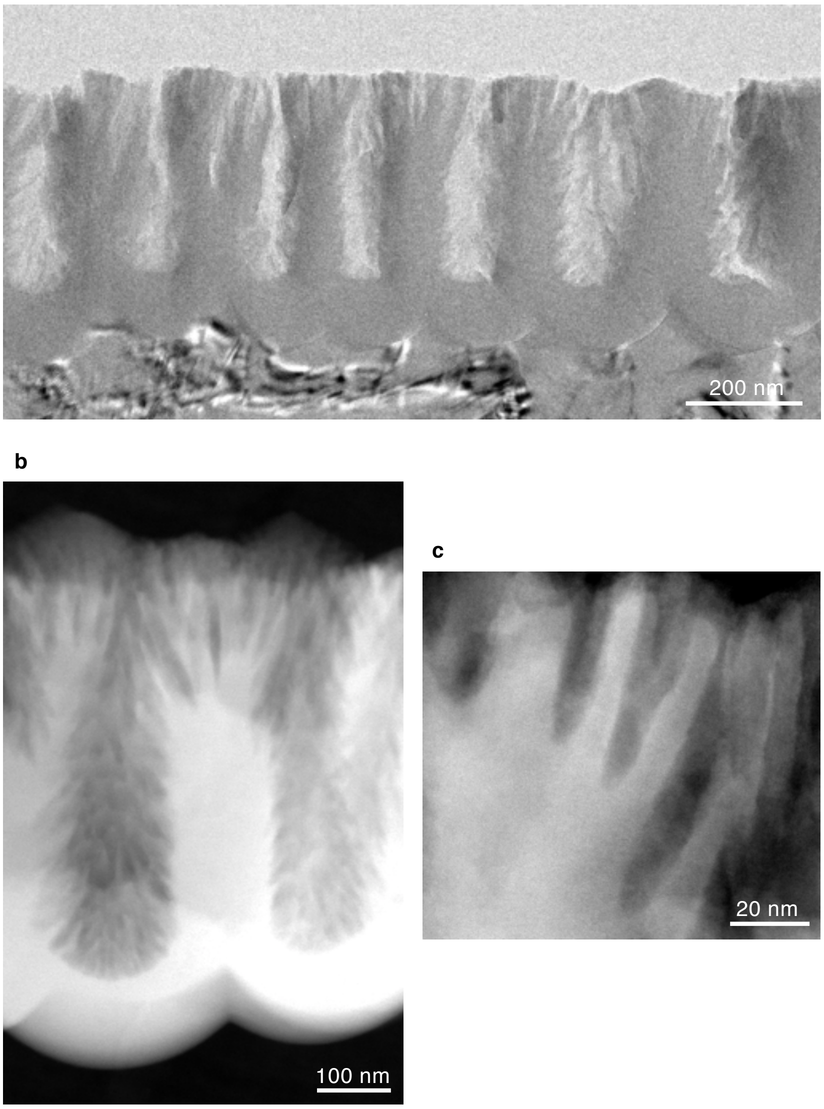

Supplementary Figure S11 a, A bright-field (BF)-STEM image of the porous AAO structure formed by anodizing in a 0.5 M sodium tetraborate solution. A continuous nanospike pore array was formed on the Al substrate. **b,** An HAADF-STEM image of the porous AAO structure. Numerous spike nanostructures were clearly formed over the whole pore wall from the top surface to the bottom interface. **c,** A high-magnification HADDF-STEM image of the pore entrance. The nanospike structure was constructed with numerous smaller pores of approximately 10 nm or less in diameter.

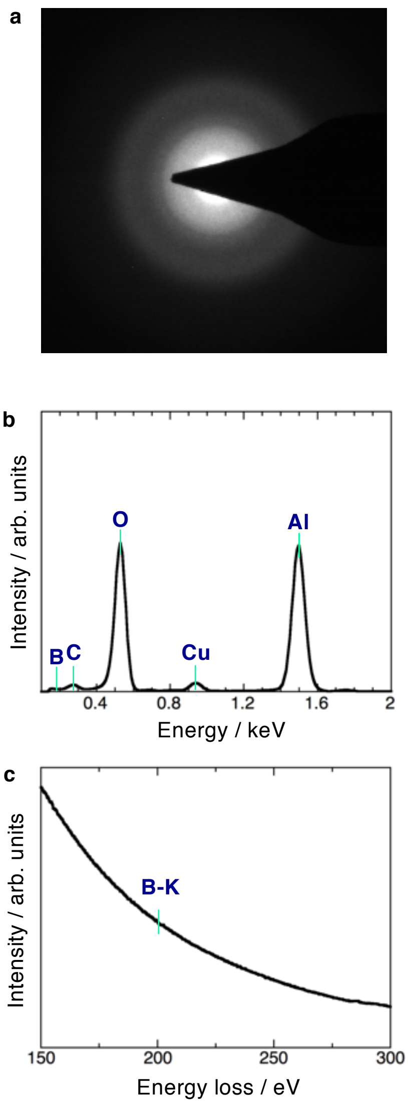

Supplementary Figure S12 a, A TEM diffraction pattern of the porous AAO film formed by the anodizing process in sodium tetraborate solution. The AAO film consisted of amorphous aluminum oxide. **b,** STEM-energy dispersive spectroscopy (EDS) spectrum of the porous AAO film. There was no peak for B, whereas four clear peaks for O and Al originated from AAO, C from contamination, and Cu from the TEM grid. **c,** Electron energy loss spectroscopy (EELS) spectrum of the porous AAO film; no B-K peak is present in the spectrum.
